# THE MANY FACES OF DEMENTIA IN CANADA: EXAMINING SEX, ETHNICITY, AND AGE OF ONSET IN THE LANDMARK STUDY

**DOI:** 10.1093/geroni/igad104.0283

**Published:** 2023-12-21

**Authors:** Joshua Armstrong, Saskia Sivananthan, Josée Guimond

**Affiliations:** Alzheimer Society of Canada, Toronto, Ontario, Canada; Alzheimer Society of Canada, Toronto, Ontario, Canada; Alzheimer Society of Canada, Toronto, Ontario, Canada

## Abstract

With the rapidly increasing size of the dementia population, it is important to enhance our understanding of the similarities and differences that are found across the people living with condition. This work takes a closer look at the many faces of dementia in Canada by highlighting the diversity that is found within the national population projections for dementia. To conduct these forecasts, a simulation model was developed using demographic characteristics and risk factors for dementia to estimate the numbers of people living with dementia in Canada from 2020-2050. From the results of the Landmark Study, we will highlight findings related to sex, Indigenous Peoples, ethnic origins, and young-onset dementia. In 2020, 61.3% of dementia diagnoses were in females and this sex ratio is projected to stay constant over the three decades. For Indigenous Peoples of Canada, dementia numbers are estimated to increase by 273% (2020: 10,800; 2050: 40,300). People of Asian origin will increase from 8% of people living with dementia in 2020 to 24% by 2050. The changes are driven both by future immigration, and by people who have already immigrated to Canada in the past few decades. The Landmark Study also projects that there could be over 40,000 people under the age of 65 living with dementia in Canada by 2050.These findings illustrate the changing landscape of people living with dementia in Canada. These demographic characteristics, as well as other distinctions across population groups, profoundly affect the way in which dementia is experienced and their care needs.

